# HER2 positivity predicts BCG unresponsiveness and adaptive immune cell exhaustion in EORTC risk-stratified cohort of bladder cancer

**DOI:** 10.3389/fimmu.2023.1301510

**Published:** 2023-12-08

**Authors:** Wook Nam, Han Kyu Chae, Yeonuk Jung, Homin Kang, Myungchan Park, Ahnryul Choi, Jong Yeon Park, Dae-Woon Eom, Sung Jin Kim

**Affiliations:** ^1^ Department of Urology, Gangneung Asan Hospital, University of Ulsan College of Medicine, Gangneung, Republic of Korea; ^2^ Department of Urology, Asan Medical Center, University of Ulsan College of Medicine, Seoul, Republic of Korea; ^3^ Department of Urology, Haeundae Paik Hospital, Inje University College of Medicine, Busan, Republic of Korea; ^4^ Department of Biomedical Engineering, College of Medical Convergence, Catholic Kwandong University, Gangneung, Republic of Korea; ^5^ Department of Pathology, Gangneung Asan Hospital, University of Ulsan College of Medicine, Gangneung, Republic of Korea

**Keywords:** Bladder neoplasm, EORTC risk, Bacillus Calmette–Gúérin, HER2, ERBB2 mutation, T-cell exhaustion, PD-L1

## Abstract

**Introduction:**

Predicting the response to Bacillus Calmette–Guérin (BCG) therapy in high-risk patients with non-muscle invasive bladder cancer (NMIBC) is crucial, as failure may necessitate interventions, such as radical cystectomy or salvage therapy. With the recent classification of genetic class 2a (which has HER2 protein abundance as its signature mutation of *ERBB2*), evaluating its prognostic role and relationship with BCG response could yield important results.

**Methods:**

This retrospective study included 160 patients with NMIBC who underwent transurethral resection of bladder tumors at Gangneung Asan Hospital between 2000 and 2013 and were stratified based on the European Organization for Research and Treatment of Cancer (EORTC) risk criteria. In addition, we analyzed a subset of 67 patients who had received BCG induction therapy to identify factors predictive of BCG treatment response. Univariate and multivariate analyses were used to assess the impact of clinicopathological factors, HER2 positivity, and EORTC risk on recurrence, progression, survival, and BCG response. Each variable’s prognostic significance was determined using the Kaplan–Meier analysis. The tumor microenvironments (TMEs) were evaluated in relation to HER2 and EORTC risk.

**Results:**

Patients with HER2+ had a higher median age, a greater prevalence of high-grade tumors, and more frequent recurrences. The univariate analysis demonstrated that the HER2+, intermediate (*vs*. low-risk) high (*vs*. low-risk), and EORTC recurrence risk groups were significantly associated with recurrence. In patients treated with BCG, only the HER2+ status predicted recurrence. In the univariate analysis for progression, age, high EORTC progression risk (*vs*. low-to-intermediate), HER2+, and programmed death-ligand 1 positive (PD-L1+) were significant factors. In multivariate analyses for progression, age, high EORTC progression risk, and PD-L1+ were significant factors for progression. HER2 expression was associated with the TME, influencing the proportion of PD-L1+ cells, as well as other markers of PD-1, CD8, and Ki67.

**Conclusion:**

The HER2+ status may be related to genetic characteristics that appear more frequently in older age, which suggests a potential for predicting the recurrence and response to BCG treatment. Additionally, analyzing TME trends of aggressive adaptive immune response characterized by HER2 expression provides insight into recurrence and BCG response mechanisms.

## Graphical Abstract

“HER2 positivity in de novo Non-Muscle Invasive Bladder Cancer”: More Common in the Elderly and Exhibiting Characteristics of an Aggressive T-cell Tumor Microenvironment Similar to Molecular Subtype Class 2a. This feature of HER2 positivity in bladder cancer predicts BCG unresponsiveness, providing a potential basis for using immune checkpoint inhibitors or HER2 therapeutics in conjunction with BCG in the initial treatment phase for BCG naive bladder cancer patients.

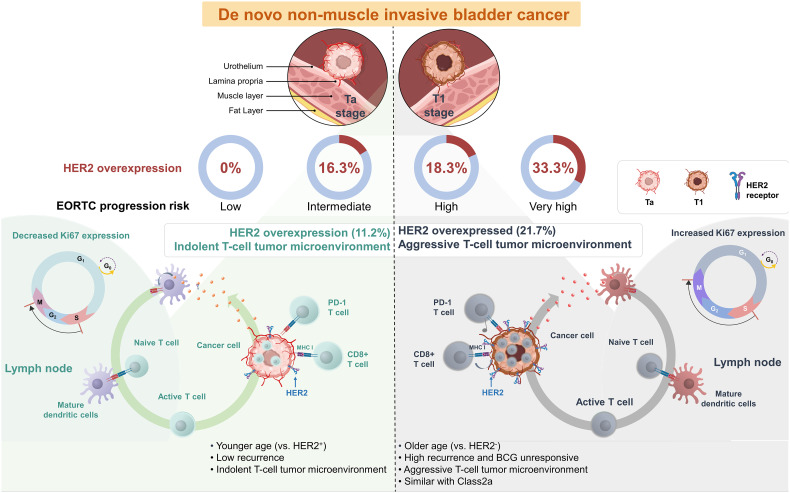

## 1 Introduction

Bladder cancer, ranked as the tenth most common malignant tumor globally, is associated with high incidence and mortality rates worldwide, with an estimated 573,278 new cases and 212,536 deaths respectively (1). Approximately 75% of patients with bladder cancer are diagnosed with non-muscle invasive bladder cancer (NMIBC) with a high risk of recurrence and poor prognosis for disease progression. Such patients are often administered adjuvant Bacillus Calmette–Guérin (BCG) treatment (2, 3). Treatment response plays a crucial role in the long-term management of the disease, as 50%–60% of patients who do not respond to initial BCG treatment are at risk of life-threatening conditions (4). High rates of disease recurrence in high-risk patients result in a heavy disease burden involving repeated transurethral resection of bladder tumor (TURBT) or radical cystectomy, which negatively impacts their quality of life (5, 6).

Identifying the risk of progression from NMIBC to muscle-invasive bladder cancer (MIBC) presents a significant clinical challenge. Therefore, to address this issue, the European Organization for Research and Treatment of Cancer (EORTC) Genito-Urinary Group developed EORTC risk tables in 2006 to predict the recurrence and progression of NMIBC (7). However, these tables have limited inclusion of patients who have undergone BCG management, leading to an overestimation of the risk of recurrence or progression. Consequently, the Spanish Urological Club for Oncological Treatment (CUETO) scoring system included many patients who received BCG treatment to overcome these limitations (8). Despite these efforts, in patients classified as high-risk, only approximately 20% have MIBC (24% and 17% for EORTC and CUETO, respectively) (7, 9). Therefore, identifying markers that can improve risk stratification and promote effective treatments for high-risk patients with NMIBC who require BCG treatment is crucial.

Accurately predicting the response to BCG therapy is crucial, particularly considering the potential to apply a subsequent therapy simultaneously as BCG naïve phase treatment (10). However, previous attempts to predict responses to BCG treatment have been unsuccessful (11). Recently, building upon the UROMOL study, four molecular subtypes were identified in NMIBC; these were stratified based on their *ERBB2* mutation (12, 13). Given the increasing emphasis on the role of HER2 in the genetic subclass, it is necessary to examine the association of HER2-positivity (HER2+) and BCG response in high-risk patients. Therefore, to investigate this, we categorized patients with NMIBC based on EORTC risk classification, EAU risk (European Association Urology risk group 2023) and evaluated the role of HER2+ in prognosis. In particular, we analyzed whether HER2+ plays an additional role compared to EORTC risk in patients who received BCG in changing the tumor microenvironment (TME).

## 2 Methods

### 2.1 Ethical approval

Ethical approval for this study was provided by the Institutional Review Board of Gangneung Asan Hospital, Gangneung, Republic of Korea (no. 2022-04-013). The requirement for informed consent was waived owing to the retrospective nature of the study.

### 2.2 Patients’ eligibility

**Figure 1:**
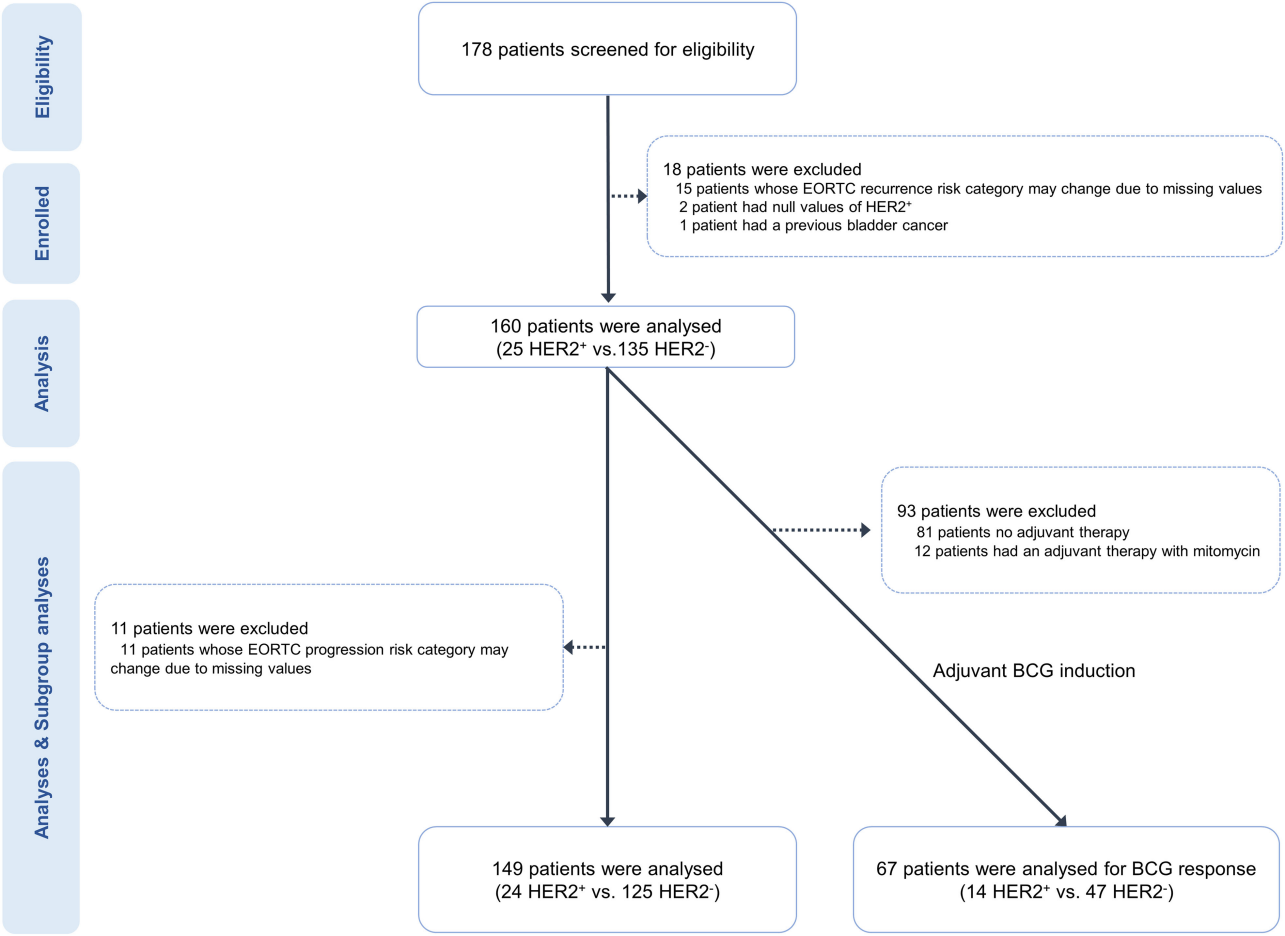
Flow chart showing patients and study design.

We analyzed 160 patients who underwent their initial bladder cancer surgery at our hospital between 2000 and 2013 (**Figure 1**). Of the 178 patients initially screened for eligibility, we excluded 15 due to missing clinical variables, making it impossible to determine their EORTC classification. Additionally, two patients could not be categorized based on HER2 expression due to staining issues, and one had a previous history of bladder cancer. We evaluated the progression-free survival (PFS) and overall survival (OS) in 149 patients with determinable EORTC progression risk group (EORTC-P), excluding 11 whose EORTC-P could not be determined. Furthermore, we conducted a subgroup analysis of 67 patients who underwent intravesical BCG therapy.

### 2.3 EORTC risk group

To evaluate the recurrence and progression of patients with NMIBC, we collected information on tumor number, tumor size, prior recurrence rate, T stage, the presence of carcinoma *in situ* (CIS), and tumor grade, as provided by the EORTC risk. Our patient cohort was categorized into four risk groups (low, intermediate, high, and very high) using the EORTC recurrence risk group (EORTC-R), EORTC-P and EAU risk. **Tables S1** and **S2** present the scoring system for recurrence and progression according to the EORTC and the corresponding notations for each risk group in this study (7).

The tumor grade was determined based on the pathological results using the World Health Organization 1973 classification, which classifies most patients into grades 1, 2, and 3 and distinguishes between low- and high-grade tumors (14). Fifteen patients were excluded from this study because their risk category could change if their EORTC-R scoring were performed using the Grades 1–3 system. Initially, these patients were categorized as either low-grade or high-grade (**Table 1**).

**Table 1:** Patient characteristics, tumor features, and outcomes based on HER2+ expression.

Number of patients, n (%)	Total	HER2+	HER2−	*p*-value
	160 (100)	25 (15.6)	135 (84.4)	
Age, year, median [IQR]	70.0 [61.0–77.0]	76.0 [69.0–79.0]	69.0 [60.0–76.5]	<0.05
Sex, n (%)				0.72
Male	129 (80.6)	19 (76.0)	110 (81.5)	
Female	31 (19.4)	6 (24.0)	25 (18.5)	
Tumor size (cm)				0.88
<3	97 (60.6)	16 (64.0)	81 (60.0)	
≥3	63 (39.4)	9 (36.0)	54 (40.0)	
Number of tumors, n (%)				0.14
Single	92 (57.5)	11 (44.0)	81 (60.0)	
2–7	63 (39.4)	14 (56.0)	49 (36.3)	
≥7	5 (3.1)	0 (0.0)	5 (3.7)	
Tumor stage, n (%)				0.10
Ta	85 (53.1)	9 (36.0)	76 (56.3)	
T1	75 (46.9)	16 (64.0)	59 (43.7)	
Tumor grade, n (%)				0.06
Low grade	49 (30.6)	3 (12.0)	46 (34.1)	
High grade	101 (63.1)	21 (84.0)	80 (59.3)	
Tumor grade loss	10 (6.2)	1 (4.0)	9 (6.7)	
Concurrent CIS, n (%)	12 (7.5)	3 (12.0)	9 (6.7)	
EORTC-R risk, n (%)				0.48
Low	12 (7.5)	0 (0.0)	12 (8.9)	
Intermediate	67 (41.9)	11 (44.0)	56 (41.5)	
High risk	74 (46.2)	13 (52.0)	61 (45.2)	
Very high	7 (4.4)	1 (4.0)	6 (4.4)	
EORTC-P risk, n (%)				<0.05
Low	28 (17.5)	0 (0.0)	28 (20.7)	
Intermediate	43 (26.9)	7 (28.0)	36 (26.7)	
High	60 (37.5)	11 (44.0)	49 (36.3)	
Very high	18 (11.2)	6 (24.0)	12 (8.9)	
EAU risk, n (%)				<0.05
Low	28 (17.5)	2 (8.0)	28 (17.5)	
Intermediate	38 (23.8)	2 (8.0)	38 (23.8)	
High	83 (51.9)	19 (76.0)	83 (51.9)	
Very high	11 (6.9)	2 (8.0)	11 (6.9)	
Adjuvant therapy				
No adjuvant therapy	81 (50.6)	15 (60.0)	66 (48.9)	0.54
Adjuvant BCG, n (%)	67 (41.9)	9 (36.0)	58 (43.0)	
Adjuvant mitomycin, n (%)	12 (7.5)	1 (4.0)	11 (8.1)	
Recurrence	101 (63.1)	21 (84.0)	80 (59.3)	<0.05
Progression	17 (10.6)	5 (20.0)	12 (8.9)	0.19
Expire event	80 (50.0)	14 (56.0)	66 (48.9)	0.66
Survival period	112.5 [54.0–146.5]	106.0 [34.0–133.0]	115.0 [70.0–156.5]	0.08

### 2.4 Definitions of recurrence, progression, and survival

The patients in this study had a standard follow-up regimen that included upper tract imaging (ultrasonography or computed tomography) once a year and a cystoscopy every 3–6 months. If any suspicious lesions were found, a biopsy or elective surgery was performed to assess the lesion, and recurrence was determined after pathological confirmation of the lesion. Progression was defined as bladder cancer recurrence with pathologically confirmed pT2 or higher lesions. In cases where pathological confirmation of pT2 was impossible, any procedures performed that were related to MIBC (such as radical cystectomy, radiation therapy, or chemotherapy) were also considered as progression. Survival data were obtained from the Korean National Health Insurance System, and all patients included in this study were registered.

### 2.5 Definition of BCG response

The response to BCG treatment in NMIBC was evaluated based on the guidelines provided by the International Bladder Cancer Group for clinical trial design (15). Treatment failure following BCG encompasses two forms of recurrence: ‘Refractory’ and ‘Relapse’. Refractory to BCG was defined as bladder cancer recurrence or persistence within 6 months despite surgical treatment and appropriate BCG therapy. Relapse to BCG was defined as cancer recurrence 6 months after receiving appropriate BCG treatment. Since the refractory disease was considered within a 3-month lead time, patients experiencing a relapse after 9 months were classified in this category.

### 2.6 Tissue microarray

Overall, 178 tumor samples were collected from TURBT specimens. The specimens were fixed in formalin and embedded in paraffin. A QuickRay tissue-arraying tool (Unitma Co., Ltd., Seoul, Korea) was used to evaluate Tissue Microarray (TMA). The typical tumor regions were identified using the stained slides with hematoxylin and eosin, and corresponding tissue blocks were collected. We used 2 mm diameter tissue cylinders to punch the areas in tumor blocks that corresponded to the identified regions, and these cores were subsequently relocated to recipient TMA blocks. Next, we sliced the TMA blocks into sections with a thickness of 4 µm for immunohistochemical (IHC) staining.

### 2.7 Immunohistochemistry

The programmed death-ligand 1 (PD-L1) protein was detected using IHC staining with two unique antibody clones (SP263, Ventana Medical Systems, Tucson, USA) on an automated staining platform (Ventana). For the IHC staining of CD8 and PD-1, we used the SP16 (Thermo Fisher Scientific, Runcorn, UK; 1:100) and the EPR4877 (Abcam, Cambridge, UK; 1:100) antibodies, respectively. These stains were applied via the Bond-Max automatic immunostaining tool (Leica Biosystems). For HER2 and Ki-67 IHC staining, we employed 4B5 (Roche Diagnostics, Tucson, USA; pre-dilution) and SP6 (Cell Marque, California, USA; 1:300) antibodies, respectively, using the same automated staining system. Placental tissue sections were positive controls for PD-L1, while tonsil tissue sections were used for PD-1, CD8, and Ki67. The primary antibodies were omitted to prepare negative controls, and a rabbit monoclonal immunoglobulin (Ventana) was used as a negative control for PD-L1 staining.

### 2.8 IHC analyses

The PD-L1 immunoreactivity was evaluated independently of the clinicopathologic details. We used membrane-associated immunostaining to assess PD-L1 expression, focusing on the percentage of definitive positive staining in the tumor epithelial and the immune cells that infiltrated the tumor, irrespective of the staining intensity. The degree of positive staining in epithelial tumor cells was classified into the following four categories: 0 (under 1%), 1 (between 1% and 5%), 2 (at least 5% but <50%), and 3 (≥50%). The proportion of infiltrating immune cells that were positively stained was similarly classified into the following four categories: 0 (<1%), 1 (at least 1% but not exceeding 5%), 2 (between 5% and 10%), and 3 (≥10%). PD-L1 expression was deemed positive if the grade of immunoreactivity was at least 1 and was further categorized as follows: 0 (absent), 1 (low), 2 (moderate), and 3 (high) (16). Immunostaining for PD-1 and CD8 was applied to the sections to determine the presence of tumor-infiltrating lymphocytes (TILs) within the tumor bed (**Additional File 1: Figures S1A, B**). We examined immune cells in each section using a microscope at 400x magnification (BX51; Olympus, Tokyo, Japan). We randomly selected five distinct areas containing TILs from each sample to ensure accurate representation and consistency. Subsequently, we documented the number of immune cells in these fields and computed the average count based on a single 200x microscopic field (0.1590 mm^2^/field). The number of nuclear Ki67-positive cells (per 1,000 cells) was manually counted in the hotspots at high magnification, and the Ki67 value was evaluated using the labeling index. The arrays were read according to the given tissue microarray map, each core was individually scored, and the results were presented as the average of three replicate core samples (17, 18). The Ki-67 value was determined using the Ki-67 labeling index (**Additional File 1: Figure S1C**).

### 2.9 HER2 expression

HER2 expression levels were evaluated in accordance with breast cancer guidelines (19). The categorizations for HER2 negative (HER2−) staining were as follows: HER2 0, indicating no staining (**Additional File 1: Figure S2A**, IHC score 0); and HER2 1+, demonstrating faint or partial membrane staining in no more than 10% of cancerous cells (**Additional File 1: Figure S2B**, IHC score 1). The HER2 positive (HER2+) staining was classified as follows: HER2 2+, showing mild or moderate complete membrane staining in more than 10% of cancerous cells (**Additional File 1: Figure S2C**, IHC score 2); and HER2 3+, indicating strong complete membrane staining in more than 10% of neoplastic cells (**Additional File 1: Figure S2D**, IHC score 3).

### 2.10 Statistical analyses

We examined the relationship between clinicopathological factors, biomarkers, EORTC classification, EAU risk and oncologic outcomes using either Pearson’s χ2 or Fisher’s exact test, and continuous variables were analyzed using independent *t*-tests. The impact of clinicopathological factors, biomarkers, EAU risk and EORTC classification on recurrence-free survival (RFS), BCG response, PFS, and OS was assessed using Kaplan–Meier models with the log-rank test. Univariate and multivariate Cox proportional hazard models (Cox model) were used to evaluate the impact of clinicopathologic factors, EAU risk and EORTC classification on oncologic outcomes (RFS, BCG response, PFS, and OS). Clinicopathologic factors, biomarkers, EAU risk and EORTC classification that were evaluated as significant factors in the univariate Cox model were used to develop models for predicting oncologic outcomes in the multivariate Cox model. All statistical analyses were performed using Prism 9.3 (GraphPad). Statistical significance was set at *p* < 0.05.

## 3 Results

### 3.1 Clinicopathological findings based on HER2+ expression

**Table 1** presents data on the clinical characteristics of the 160 patients diagnosed with NMIBC. The HER2+ group exhibited a significantly higher median age than the HER2− group (*p* < 0.05). Both groups demonstrated similar tumor sizes, numbers, and stages as per the EORTC classification. However, the HER2+ group showed a higher prevalence of high-grade tumors. No significant difference was observed in the EORTC-R score based on HER2 status, although a greater proportion of patients with HER2+ had EORTC-P scores classified as high risk. A greater proportion of patients with HER2+ were classified as high risk according to the EAU risk scores. Adjuvant therapy was not performed differently between the two groups. Recurrence was more frequent in patients with HER2+ during the observation period (*p* < 0.05); however, no significant difference was found in observed progression or events. The median survival period did not significantly differ between the groups, with follow-up durations of 106 and 115 months, respectively (*p* = 0.08).

### 3.2 Clinicopathological results in patients with HER2+ following BCG treatment

The clinical and pathological outcomes of the 67 patients who underwent BCG induction therapy are summarized in **Additional File 1: Table S3**, and TME markers based on their response to BCG treatment (**Additional File 1: Table S4**). The patients in the HER2+ group exhibited a higher number of tumors than those in the HER2− group. However, no significant differences were observed regarding tumor size, stage, grade, or the presence of concomitant CIS. Although patients in the HER2+ group demonstrated a higher EORTC-R score (*p* = 0.79) and a tendency towards more frequent recurrences (*p* = 0.08) than those in the HER2+ group, these differences did not reach statistical significance.

### 3.3 Predictive factors for RFS

**Figure 2:**
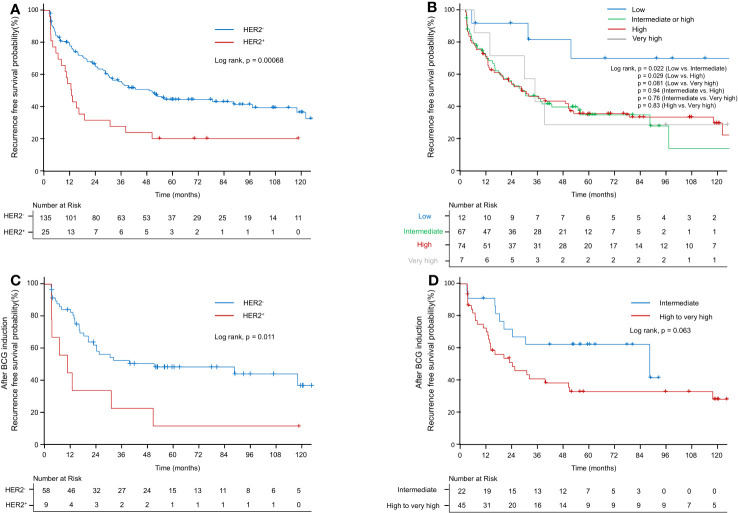
Kaplan–Meier curve of recurrence-free survival probability. **(A)** Kaplan–Meier curve of recurrence-free survival probability according to HER2+ in the EORTC-R-stratified-patient group. **(B)** Kaplan–Meier curve of recurrence-free survival probability according to EORTC-R. **(C)** Kaplan–Meier curve of recurrence-free survival probability according to HER2+ after BCG therapy. **(D)** Kaplan–Meier curve of recurrence-free survival probability according to EORTC-R after BCG therapy.

**Table 2** shows the results of the univariate analysis for RFS in the total cohort of 160 patients and a subset of 67 who received BCG induction therapy. High grade (hazard ratio [HR]: 1.593; confidence interval [CI]: 1.002–2.533; *p* < 0.05), intermediate EORTC-R score (HR: 3.002; CI: 1.069–8.431; *p* = 0.037), high EORTC-R score (HR: 2.972; CI: 1.068–8.27; *p* < 0.05), and HER2+ status (HR: 2.27; CI: 1.396–3.693; *p* < 0.01) were identified as significant risk factors for recurrence. In the subgroup of 67 patients who received BCG therapy, HER2+ status (HR: 2.682; CI: 1.223–5.882; *p* < 0.05) was found to be a risk factor for recurrence. Therefore, we attempted to construct multivariate models to predict recurrence in both groups; however, these models did not enhance the predictive accuracy beyond that of the univariate analysis. Kaplan–Meier analyses revealed HER2+ status (*p* < 0.001, **Figure 2A**), intermediate risk (compared to low risk, *p* < 0.05, **Figure 2B**), and high risk (compared to low risk, *p* < 0.05; **Figure 2B**) as significant predictors of RFS. In the BCG-treated group, HER2+ status (*p* < 0.05; **Figure 2C**) and high-to-very high risk (compared to intermediate risk, *p* = 0.06; **Figure 2D**) were identified as prognostic factors for recurrence.

**Table 2:** Univariate analysis of recurrence-free survival in all patients and those treated with BCG.

	Univariate (All patients)		Univariate (After BCG)	
	HR (95% CI)	*p*-value	HR (95% CI)	*p*-value
Age (continuous)	1.016 (0.998–1.035)	0.08	0.997 (0.963–1.032)	0.86
Sex	0.704 (0.406–1.221)	0.21	0.61 (0.237–1.568)	0.31
Adjuvant therapy				
Adjuvant therapy	0.717 (0.481–1.067)	0.10		
BCG therapy (*vs*. none)	0.776 (0.515–1.169)	0.23		
Mitomycin (*vs*. none)	0.413 (0.15–1.14)	0.09		
Pathological results				
T1 stage (*vs*. Ta)	1.281 (0.866–1.895)	0.21	1.126 (0.581–2.181)	0.73
High grade (*vs*. Low)	1.593 (1.002–2.533)	<0.05	1.692 (0.597–4.797)	0.32
Concurrent CIS	1.045 (0.505–2.159)	0.91	1.12 (0.433–2.898)	0.82
EORTC-R risk				
Low	reference			
Intermediate (*vs*. low)	3.002 (1.069–8.431)	<0.05	Reference	
High (*vs*. low)	2.972 (1.068–8.270)	<0.05	2.096 (0.977–4.5)	0.06
Very high (*vs*. low risk)	2.695 (0.719–10.093)	0.14	1.485 (0.4–5.51)	0.55
Tumor Microenvironment				
HER2+	2.27 (1.396–3.693)	<0.01	2.682 (1.223–5.882)	<0.05
PD-L1^+^	1.002 (0.674–1.491)	0.99	1.075 (0.561–2.062)	0.82

### 3.4 Predictive factors for PFS

**Figure 3:**
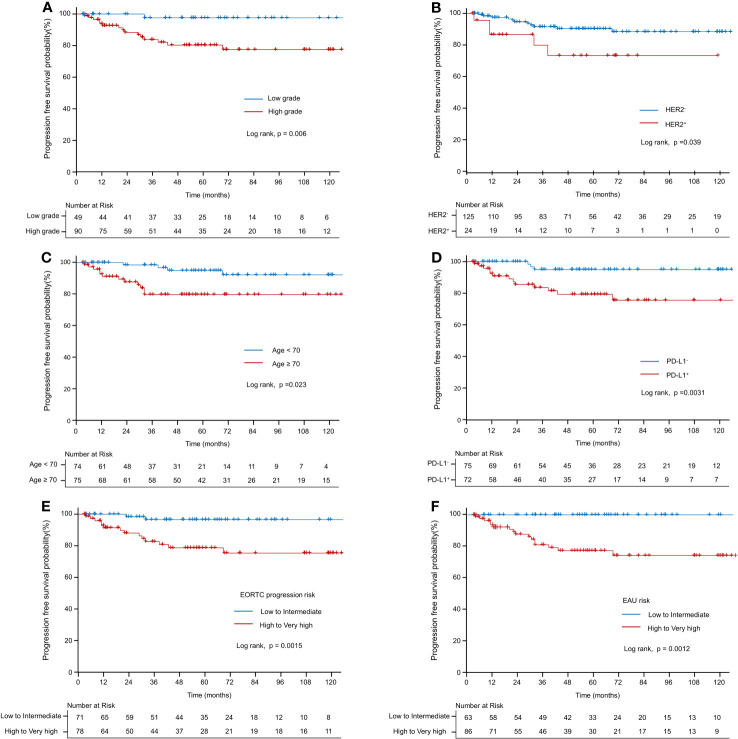
Kaplan–Meier curve of progression-free survival probability. **(A)** Kaplan–Meier curve of progression-free survival probability according to grade. **(B)** Kaplan–Meier curve of progression-free survival probability according to HER2+. **(C)** Kaplan–Meier curve of progression-free survival probability according to old age (≥70 *vs*. <70). **(D)** Kaplan–Meier curve of progression-free survival probability according to PD-L1. **(E)** Kaplan–Meier curve of progression-free survival probability according to EORTC-P (low to intermediate risk *vs*. high-to-very-high risk). **(F)** Kaplan–Meier curve of progression-free survival probability according to the EAU2023 risk (low to intermediate risk *vs*. high to very high risk).

**Table 3** presents the results of univariate and multivariate analyses for PFS. In the univariate analysis of the entire patient cohort (n = 149), several significant factors were associated with progression. These factors included age (HR: 1.069; CI: 1.022–1.119; *p* < 0.05), high grade (HR: 9.968; CI: 1.316–75.518; *p* = 0.05), EORTC-P (HR: 7.628; CI: 1.733–33.583; *p* < 0.01), EAU risk (HR: 5×10^9^; CI: 0.000–∞, *p* < 0.001), HER2+ status (HR: 2.912; CI: 1.01–8.395; *p* < 0.05), and PD-L1^+^ status (HR: 5.399; CI: 1.538–18.958; *p* < 0.01). Kaplan–Meier analyses revealed that high grade (*vs*. low grade, *p* < 0.001, **Figure 3A**), HER2+ status (*vs*. HER2−, *p* < 0.05; **Figure 3B**), age <70 (*vs*. age ≥70, *p* < 0.05; **Figure 3C**), PD-L1^+^ status (*vs*. PD-L1-, *p* < 0.05; **Figure 3D**), high-to-very-high risk (*vs*. low-to-intermediate risk, *p* < 0.01; **Figure 3E**) were significant predictors of progression and high-to-very-high risk (*vs*. low-to-intermediate risk, *p* < 0.01; **Figure 3F**) were significant predictors of progression. In the multivariate analysis, the optimal model (Model 1, **Table 3**) included age (HR: 1.064; CI: 1.01–1.121; *p* = 0.0196), EORTC-P (HR: 5.07; CI: 1.131–22.718; *p* = 0.0339), and PD-L1^+^ status (HR: 3.76; CI: 1.056–13.391; *p* = 0.041). Model 2 (**Table 3**) incorporated HER2+; however, adding HER2+ did not improve the model’s predictive power, and its significance was lost due to other variables (HR: 1.385; CI: 0.461–4.159; *p* = 0.5611).

**Table 3:** Univariate and multivariate analyses for progression-free survival and overall survival.

Variables	Univariate (PFS)			Univariate (survival)	
	HR (95% CI)	*p*-value	Variables	HR (95% CI)	*p*-value
Age (continuous)	1.069 (1.022–1.119)	<0.01	Age (continuous)	1.09 (1.065–1.116)	<0.001
Sex	0.907 (0.258–3.185)	0.88	Sex	1.093 (0.610–1.958)	0.77
Adjuvant therapy	0.688 (0.250–1.895)	0.47	Adjuvant therapy	0.466 (0.286–0.760)	<0.01
Pathological results			Pathological results		
T1 stage (*vs*. Ta)	NA	NA	T1 stage (*vs*. Ta)	2.393 (1.491–3.841)	<0.001
High grade (*vs*. Low)	9.968 (1.316–75.518)	<0.05	High grade (*vs*. Low)	2.035 (1.158–3.576)	<0.05
Concurrent CIS	0.761 (0.100–5.769)	0.79	Concurrent CIS	1.195 (0.545–2.620)	0.66
EORTC-P low to intermediate risk	reference		EORTC-P low to intermediate risk		
EORTC-P high risk	7.628 (1.733–33.583)	<0.01	EORTC-P high risk	1.902 (1.177–3.073)	<0.01
EAU low to intermediate risk	reference		EAU low to intermediate risk	reference	
EAU high to very high risk	5×10^9^ (0.000–∞)	<0.001	EAU high to very high risk	2.641 (1.576–4.424)	<0.001
Tumor microenvironment			Tumor microenvironment		
HER2+ a	2.912 (1.010–8.395)	<0.05	HER2+ a	1.631 (0.910–2.923)	0.10
PD-L1^+^ a	5.399 (1.538–18.958)	<0.01	PD-L1^+^ a	1.18 (0.742–1.877)	0.49
Multivariate analysis			Multivariate analysis		
Model 1			Model 4		
Age	1.064 (1.010–1.121)	<0.05	Adjuvant therapy	0.334 (0.201–0.552)	<0.001
EORTC-P high	5.07 (1.131–22.718)	<0.05	EAU high to very high risk	3.584 (2.101–6.114)	<0.001
PD-L1^+^	3.76 (1.056–13.391)	<0.05	Model 5		
Model 2			Age	1.079 (1.054–1.106)	<0.001
Age	1.063 (1.008–1.120)	<0.05	Adjuvant therapy	0.37 (0.218–0.628)	<0.001
EORTC-P high risk	4.885 (1.081–22.085)	<0.05	EORTC-P high risk	2.026 (1.201–3.416)	<0.01
HER2+	1.385 (0.461–4.159)	0.56	HER2+	1.14 (0.631–2.059)	0.67
PD-L1^+^	3.487 (0.947–12.839)	0.06			

### 3.5 Predictive factors for OS

**Figure 4:**
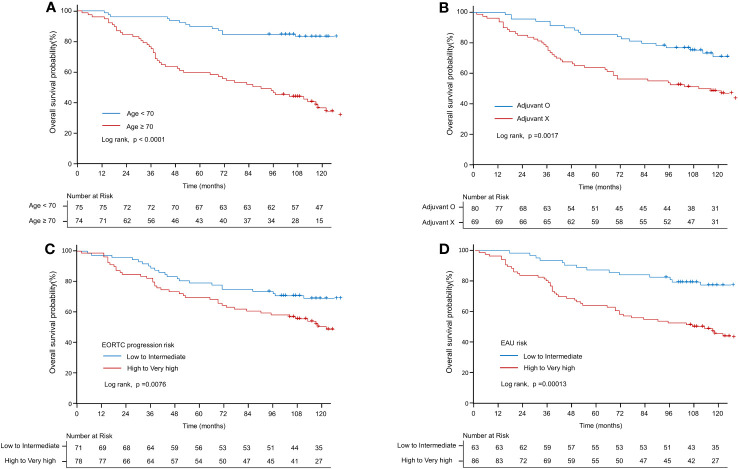
Kaplan–Meier curve of overall survival probability. **(A)** Kaplan–Meier curve of OS probability according to old age (≥70 *vs*. <70 years). **(B)** Kaplan–Meier curve of OS probability according to adjuvant therapy. **(C)** Kaplan–Meier curve of OS probability according to EORTC-P (low to intermediate risk *vs*. high to very high risk). **(D)** Kaplan–Meier curve of OS probability according to the EAU2023 risk (low to intermediate risk *vs*. high to very high risk).

**Table 3** presents the results of both univariate and multivariate survival analyses. According to the univariate analysis, age demonstrated a significant association with survival (HR: 1.09; CI: 1.065–1.116; *p* < 0.001). However, no significant association was observed between sex and survival (HR: 1.093; CI: 0.61–1.958; *p* = 0.765). Adjuvant therapy positively impacted survival (HR: 0.466; CI: 0.286–0.76; *p* < 0.01). Pathological factors, such as T1 stage (compared to Ta) (HR: 2.393; CI: 1.491–3.841; *p* < 0.001) and high-grade tumors (compared to low-grade) (HR: 2.035; CI: 1.158–3.576; *p* < 0.05), were found to be significant predictors of survival. However, the presence of concurrent CIS was not significantly associated with survival (HR: 1.195; CI: 0.545–2.62; *p* = 0.657). Moreover, patients classified as high-risk according to the EORTC-P risk and EAU risk exhibited a significantly higher risk of mortality than those classified as low to intermediate risk, respectively (HR: 1.902; CI: 1.177–3.073; *p* < 0.01), (HR: 2.641; CI: 1.576–4.424; *p* < 0.001). Regarding microenvironmental markers, HER2 and PD-L1 showed no significant association with survival in the univariate analysis. Kaplan–Meier analyses revealed that age <70 (compared to age ≥70, *p* < 0.001; **Figure 4A**), adjuvant therapy (compared to no adjuvant therapy, *p* < 0.01, **Figure 4B**), high-to-very-high risk EORTC-P (compared to low-to-intermediate risk, *p* < 0.01; **Figure 4C**) and high-to-very-high risk EAU risk (compared to low-to-intermediate risk, p < 0.01; **Figure 4D**) were significant predictors of survival. In the multivariate analysis, the optimal model (Model 3, **Table 3**) included age (HR: 1.079; CI: 1.054–1.105; *p* < 0.001), adjuvant therapy (HR: 0.368; CI: 0.217–0.625; *p* < 0.001), and EORTC-P (HR: 2.06; CI: 1.229–3.454; *p* < 0.01) as predictors. Model 4 (**Table 3**) included adjuvant therapy (HR: 0.334; CI: 0.201–0.552; *p* < 0.0001) and EAU risk (HR: 3.584; CI: 2.101–6.114; *p* < 0.0001) as predictors. Model 5 (**Table 3**) incorporated HER2+ into Model 3; however, adding HER2+ did not improve the model’s predictive power, and its significance was reduced due to other variables (HR: 1.14; CI: 0.631–2.059; *p* = 0.67).

### 3.6 EORTC-R, EORTC-P, and HER2 expression: impact on the TME

**Figure 5:**
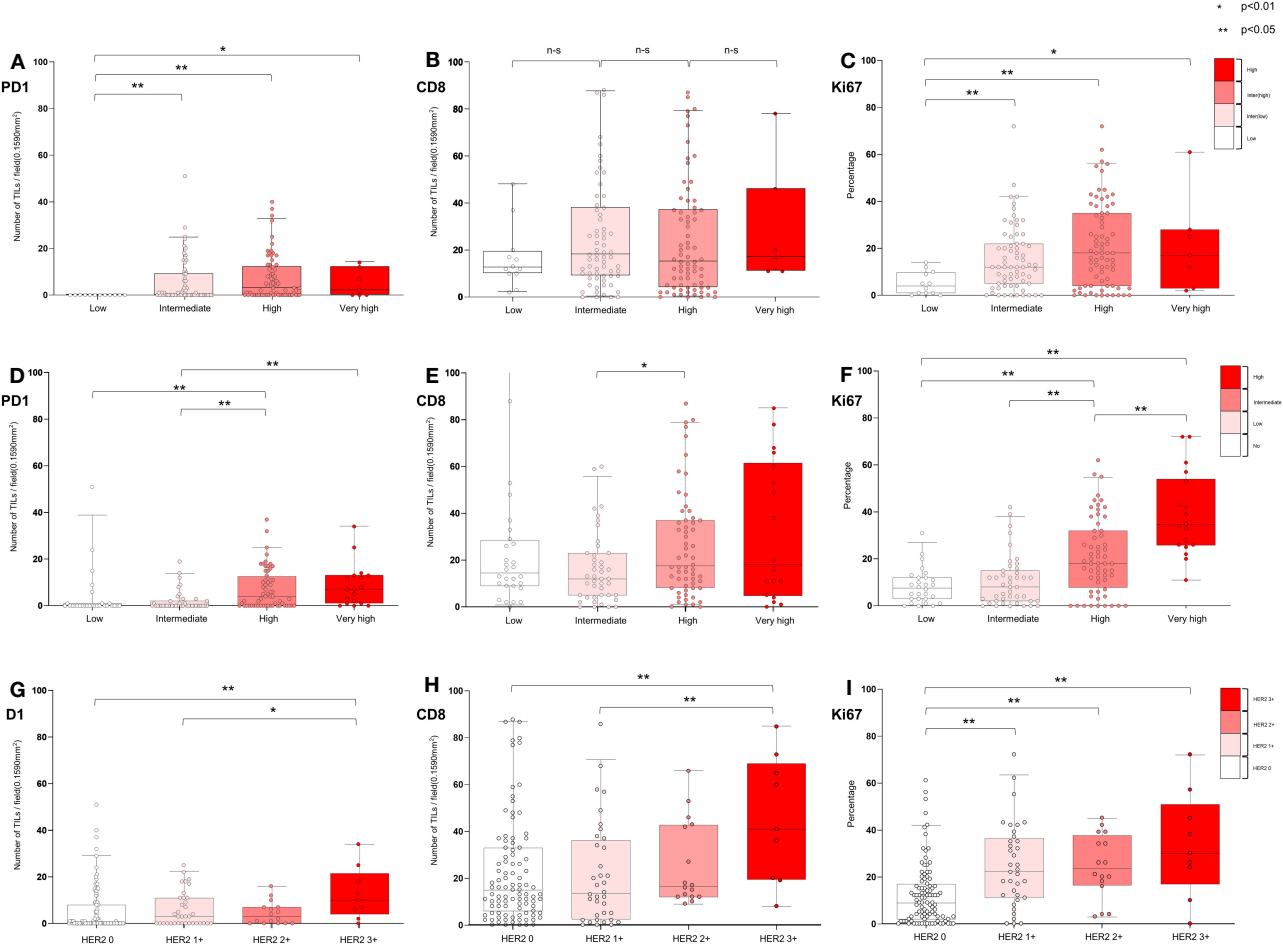
Characterization of tumor microenvironment according to EORTC risk and HER2 expression in all patients. **(A−C)** Association of EORTC-R risk groups and tumor microenvironment **(D−F)** Association of EORTC-P risk groups and tumor microenvironment **(G−I)** Association of HER2+ and microenvironment. **(A−I)** Box plots showing tumor-infiltrating lymphocytes per field (0.1590 mm2) (PD-1 and CD8) and growth fraction of cells (Ki-67) Box plots indicate the bottom, upper and lower quartiles. The middle bands indicate the median and whiskers extend to the 95th percentile. Each value is displayed on the box and whiskers plot, and for cases with a *p*-value, *p* < 0.05 values are presented; for non-significant values, the smallest p-value is displayed. HER2+ included HER2 2+ and HER2 3+, and HER2- included HER2 0 and HER2 1+.

**Figures 5A–C** and **Additional File 1: Table S5** present the distribution of various biomarkers in 160 patients, classified according to their EORTC-R group as follows: low (n = 12), intermediate (n = 67), high (n = 74), and very high (n = 7). No significant difference was found in the frequency of HER2 expression between the low-to-intermediate and high-to-very-high-risk groups (*p* = 0.71). However, significant differences were observed in the levels of PD-1 (*p* < 0.01), CD8 (*p* < 0.05), and Ki67 (*p* < 0.001) among the groups. High-to-very-high-risk patients exhibited higher levels of these biomarkers than low-to-intermediate-risk patients. No significant difference was found in PD-L1^+^ expression between the overall risk groups (*p* = 0.527); however, a significant difference was observed when comparing subcategories (*p* < 0.05).

**Figures 5D–F** and **Additional File 1: Table S6** shows the distribution of biomarkers (HER2+, PD-L1^+^, PD-1, CD8, and Ki67) in 149 patients categorized into the following EORTC-P groups: low (n = 28), intermediate (n = 43), high (n = 60), and very high (n = 18). The results indicated a marginally significant difference in HER2+ between the low-to-intermediate and high-to-very-high-risk groups (*p* = 0.08), with a significant difference observed within subcategories (*p* < 0.05). A significant difference was observed in PD-L1^+^ expression between the risk groups (*p* < 0.05) and within the subcategories (*p* < 0.05). Notably, PD-1 (*p* < 0.001), CD8 (*p* < 0.05), and Ki67 (*p* < 0.001) levels also significantly differed between the risk groups. High-to-very-high-risk patients exhibited higher levels of biomarkers than low-to-intermediate-risk patients. **Figures 5G–I** and **Additional File 1: Table S7** present the distribution of biomarkers in 160 patients, classified based on their HER2 status [HER2^-^ (n = 135), HER2+ (n = 25)], and further categorized based on HER2 expression [HER2 0 (n = 99), HER2 1+ (n = 36), HER2 2+ (n = 16), and HER2 3+ (n = 9)]. Significant differences were observed in PD-L1^+^ status between the HER2- and HER2+ groups (*p* < 0.05) and among the categories of HER2 expression (*p* < 0.05). HER2+ tumors had a higher proportion of PD-L1^+^ cells. Additionally, patients with HER2+ exhibited significantly higher levels of PD-1 (*p <* 0.05), CD8 (*p <* 0.05), and Ki67 (*p* < 0.001) than those with HER2-, a trend that persisted across the categories of HER2 expression. These findings indicated a correlation between HER2 expression and TME characteristics, including the presence of PD-L1^+^ cells and increased levels of PD-1, CD8, and Ki67.

### 3.7 EORTC-R, EORTC-P, and HER2 expression: impact on the TME in patients treated with BCG

**Figure 6:**
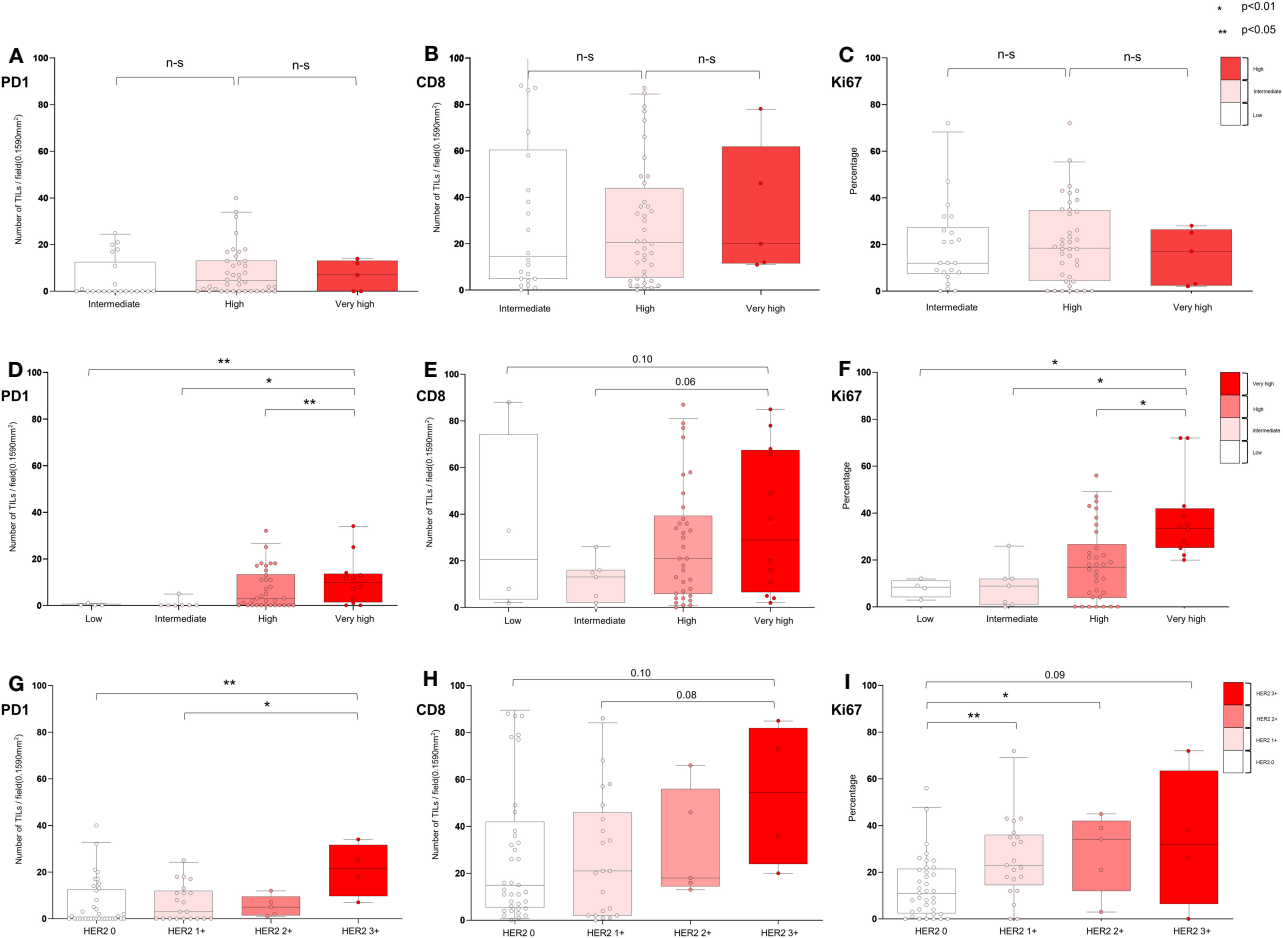
Characterization of tumor microenvironment according to EORTC risk and HER2 expression in BCG-treated patients. **(A−C)** Association of EORTC-R risk groups and tumor microenvironment after BCG therapy. **(D−F)** Association of EORTC-P risk groups and tumor microenvironment after BCG therapy. **(G−I)** Association of HER2+ expression and tumor microenvironment after BCG therapy. **(A−C)** Box plots showing tumor-infiltrating lymphocytes per field (0.1590 mm2) (PD-1, CD8) and growth fraction of cells (Ki-67). **(A−I)** Box plots showing tumor-infiltrating lymphocytes per field (0.1590 mm^2^) (PD-1, CD8) and growth fraction of cells (Ki-67) Box plots indicate the bottom, upper and lower quartiles. The middle bands indicate the median and whiskers extend to the 95th percentile. Each value is displayed on the box and whiskers plot, and for cases with a *p*-value, *p* < 0.05 values are displayed; for non-significant values, the smallest *p*-value is displayed. HER2+ included HER2 2+ and HER2 3+, and HER2− included HER2 0 and HER2 1+.

**Figures 6A–C** and **Additional File 1: Table S8** show the distribution of TME markers (PD-1, CD8, and Ki67) in 67 patients who received BCG treatment, classified into the following EORTC-R groups: intermediate (n = 22), high (n = 40), and very high (n = 5). Although not statistically significant (*p* = 0.0789), the prevalence of HER2+ appeared to be higher in the high (20.0%) and very high (20.0%) recurrence risk groups than in the intermediate group (0.0%). No significant differences were observed in median levels of PD-1 (*p* = 0.19), CD8 (*p* = 0.85), or Ki67 (*p* = 0.83) across the risk groups. These findings suggest that, in BCG-treated patients, the TME markers PD-1, CD8, and Ki67 exhibit no clear association with EORTC-R.

**Figures 6D–F** and **Additional File 1: Table S9** show the distribution of TME markers (PD-1, CD8, and Ki67) in 57 patients who received BCG treatment, classified into the following EORTC-P groups: low (n = 4), intermediate (n = 7), high (n = 34), and very high (n = 12). Although not statistically significant, the frequency of HER2+ was higher in the high-to-very-high-risk group (17.4%) than in the low-to-intermediate group (0.0%, *p* = 0.195). Median levels of PD-1 showed a significant difference (*p* < 0.01), with higher levels observed in the high-to-very-high-risk group. CD8 median levels did not significantly differ between the groups (*p* = 0.169). Ki67 median levels were higher in the high-to-very-high-risk group (*p* < 0.05). These findings indicate that the TME markers PD-1 and Ki67 may be associated with EORTC-P in BCG-treated patients.

**Figures 6G–I** and **Additional File 1: Table S10** present the distribution of TME markers (PD-1, CD8, and Ki67) in 57 patients who received BCG treatment, categorized based on the following HER2 expression levels: HER2 0 (n = 37), HER2 1+ (n = 21), HER2 2+ (n = 5), and HER2 3+ (n = 4). The analysis revealed that PD-1 expression was significantly higher in patients with HER2+ (*p* < 0.01), with the highest PD-1 expression observed in those with HER2 3+ (*p* < 0.01). Higher Ki67 expression was detected in patients with higher HER2 expression (*p* < 0.05).

## 4 Discussion

This study aimed to investigate the risk of recurrence and progression in NMIBC based on HER2+ expression, stratified based on the EORTC risk groups, and analyze the TME accordingly. HER2+ status not only distinguishes patient groups with a higher risk of recurrence in the overall patient population but also predicts treatment responses related to BCG therapy. HER2+ status was superior to the EORTC risk scoring in relation to responses to BCG treatment. Furthermore, a tendency associated with an aggressive adaptive TME was analyzed according to the HER2 expression.

Predicting BCG treatment outcomes in NMIBC is crucial, as the failure of such treatment leaves patients with limited therapeutic options, such as radical cystectomy or salvage therapy, which can significantly impair their quality of life (4–6). However, the predictive capability for progression in patients categorized as high-risk based on the EORTC or CUETO scoring systems is only approximately 20% (7, 9). In our study, among 67 patients treated with BCG, those classified with HER2+ status demonstrated a markedly poor response to BCG, and eight of the nine (88.8%) did not respond to BCG, and half of these non-responders presented as BCG refractory. HER2 expression, which is known to be genetically associated with *ERBB2* mutations and related to specific genetic classifications, provides an opportunity to explore its correlation with these genetic subgroups (20, 21). The UROMOL genetic classification categorizes NMIBC into three classes, with Class 2 encompassing aggressive tumors that frequently recur (11, 12). In a recent classification based on UROMOL, NMIBC was further subcategorized into four subgroups, with Class 2a characterized by the presence of an *ERBB2* mutation, potentially linking the HER2+ group. The HER2+ group shares several characteristics with Class 2a, including late cell cycle (high Ki67 percentage), MHC class 1 (CD8+ T cell infiltration), PD-1 T cell infiltration, and high recurrence rates (13). These characteristics may contribute to the high recurrence rates observed in HER2+ tumors (22).

This finding is associated with the activation of adaptive immune responses, and a high proportion of late-cycle cells indicates an aggressive immune environment linked to relapse in a clinically relevant patient group. The increased density of activated T cells (PD-1 and CD8) in HER2+ cases may reflect an aggressive immune state characterized by allocating limited T cell resources. Since the primary therapeutic effect of BCG relies on its anticancer mechanism through T cell infiltration into tumor cells, a substantial portion of the available T cell resources is already activated. Consequently, the potential for additional T-cell mobilization through BCG treatment is limited (20, 22). HER2+ status could be a predictive biomarker for recurrence and BCG response, highlighting an aggressive biological TME. This immunological connection between patients with HER2+ higher relapse rates and diminished response to BCG treatment warrants further investigation.

Evaluating a mechanism of BCG therapy has played a pivotal role for several decades in NMIBC, leading to attempts to better understand the associated responses and antitumor mechanisms (21, 23). However, due to the activation of multiple major immune pathways by BCG treatment, identifying the primary immune activity responsible for its therapeutic effects has proven to be a complex task (24, 25). Recent studies have focused on T cells and immune checkpoints linked to relapse and BCG treatment failure, providing insights into these mechanisms (26–28). Higher PD-L1 expression has been observed in BCG granulomas of patients with NMIBC, offering preliminary evidence of a relationship between T-cell immunity, BCG treatment, and T-cell depletion associated with BCG failure (29). Aggressive TMEs have been shown to be associated with increased NMIBC recurrence (30). Our findings suggest that HER2 is a predictor not only for BCG treatment failure but also aligns with the molecular characteristics identified in recent molecular classifications with aggressive T-cell immune environments. Moreover, these results elucidate the mechanisms underlying BCG failure and provide insights into the basis for using immune checkpoint inhibitors (ICIs) in BCG-failed patients.

Recently, ICI has been approved for high-risk patients with NMIBC who do not respond to BCG (25). Although ICIs have demonstrated high efficacy in certain patients, their clinical application is limited due to variable response rates and significant side effects. Therefore, biomarkers are needed to identify patients who would benefit from ICI treatment. Our findings indicate similarities between HER2+ and BCG-unresponsive patients, as these two groups frequently overlap. The observed characteristics in these patients, such as a high proportion of activated T-cell infiltration and elevated PD-L1 expression, not only serve as markers for BCG treatment but also potential indications for ICI use (e.g., pembrolizumab) in BCG-unresponsive patients. This not only provides additional evidence for the mechanisms underlying ICIs in BCG-unresponsive patients but also suggests that HER2+ status can be a potential biomarker for ICI use (10). Moreover, these findings indicate that patients with HER2+ may be in a state resembling immunocompromised status, making them candidates for combined BCG and ICI therapy instead of BCG alone, particularly for BCG naïve patients at a high risk of BCG failure. Given its unique properties, the HER2 antigen may even serve as a direct treatment target, thereby providing a rationale for applying HER2-targeted therapies in the context of NMIBC.

Our study demonstrated a higher prevalence of older patients presenting with HER2+ tumors, with both age and HER2 status identified as risk factors for disease progression in univariate analysis. However, age emerged as the sole significant factor in the multivariate analysis, suggesting that HER2+ status does not independently predict progression. Interestingly, the TME, which is characterized by an immunocompromised status assessed through PD-L1 expression, was identified as an independent factor for progression in both univariate and multivariate analyses, alongside age (11). These findings suggest that molecular subtypes associated with an immunocompromised TME are more likely to be linked to progression than HER2+. Nonetheless, the significant association observed between EORTC classification, progression, and survival underscores the predictive relevance of EORTC-P. Our results highlight the importance of age as a risk factor for bladder cancer progression and suggest that HER2+ status does not independently predict poor prognosis. Instead, HER2+ status could represent a genetic subclass more commonly observed in older patients, potentially associated with an aggressive TME characterized by an immunocompromised status frequently reported in this age group (31). Our study strongly indicates the significance of age as a determinant of bladder cancer progression, which is consistent with previous reports indicating a more aggressive TME in older patients (31). We propose that the HER2+ group exhibits characteristics that are frequently observed in this age group, and older patients are associated with an increased risk of recurrence. Investigating the correlations among HER2+, genetic alterations, TME, and old age is necessary to determine how HER2+ status contributes to the highly recurrent phenotype commonly observed in older patients. Therefore, further research should focus on elucidating the complex relationship between HER2+, genetic alterations, TME, and old age to develop personalized therapeutic strategies.

HER2 studies on the prognostic significance of HER2+ status in NMIBC cancer have primarily focused on comparing pre- and post-progression pathological results. These studies, which used samples only from patients who had experienced disease progression, demonstrated that HER2+ was present even before progression, as observed in comparisons between MIBC and pre-invasive bladder cancer samples (32, 33). For example, Chen et al. reported that HER2+ was already evident in many cases of patients who eventually progressed to MIBC when matched with pre-progression NMIBC samples (33). Similarly, Latif et al. found a high rate of HER2+ (76%, 19/25) in patients who progressed from NMIBC to MIBC (32). These findings indicate that genetic changes in HER2 expression occur before disease progression (27). In our study, we further confirmed previous findings of HER2+ before progression and discovered its association with long-term prognosis when expressed initially. The result that none of the 28 patients classified as low-risk according to the EORTC-P were HER2- suggests a high likelihood of HER2 expression contributing to the degree of aggressiveness. This not only assists in distinguishing low-risk patients but also contributes to risk stratification in overall patients. Notably, it provides the advantage of predicting the response to BCG treatment in aggressive NMIBC.

We focused solely on primary NMIBC, acknowledging that generalization might be challenging when including patients with recurrent cases. Therefore, we compared the HER2+ rate in bladder cancer with the generally reported HER2+ rate. The HER2+ rate was reported to be 12.4% (59/475) in studies without staging information (34). In primary NMIBC, HER2 overexpression rates are reported to be between 6–17% (35). In our study, HER2+ was observed in 15.6% (25/160) of cases, aligning with the general prevalence reported for NMIBC. In a recent study, the HER2+ rate was reported to be 35.7% in a patient group treated with BCG (36). Although this study did not categorize NMIBC in accordance with risk groups, making a direct comparison challenging, it can be inferred that these patients predominantly belong to high-risk categories based on treatment guidelines (37). In our study, HER2+ was observed in 21.7% (17/78) of high to very-high-risk patients and 33.3% (6/18) of patients in the very-high-risk group. These findings suggest that our study population does not significantly deviate from the typical HER2+ patterns associated with risk groups. The comparatively lower incidence of HER2+ in our BCG group, relative to that observed in external high-risk groups (35.7%) (36), may be attributed to stricter changes in treatment guidelines, i.e., BCG treatment being limited to high-risk groups over time (37). The actual HER2+ rate, as a biomarker for identifying relevant patients administered BCG treatment, was observed to be up to about 30%. Additionally, the absence of HER2+ in the low-risk group within our study suggests the potential for its use as a biomarker to exclude low-risk NMIBC.

The results of the present study indicate that HER2+ in NMIBC not only predicts non-responsiveness to BCG therapy but also provides a rationale for combining it with immune checkpoint inhibitors (ICIs) or Disitamab Vedotin (DV, RC48-ADC), a HER2-targeted therapeutic. Despite the use of monoclonal antibodies (trastuzumab and pertuzumab) and HER2-targeting tyrosine kinase inhibitors (lapatinib, neratinib, and afatinib) as targeted therapies for HER2-positive bladder cancer, these single agents have failed to elicit satisfactory responses (35). However, DV is an innovative humanized anti-HER2 antibody conjugated to monomethyl auristatin E through a cleavable linker; it has demonstrated impressive efficacy in metastatic urothelial carcinoma, showing an overall response rate of 51.2% and a median PFS of 6.9 months (38). The absence of grade 4 and 5 toxicities in the safety profile of DV demonstrates its potential applicability for treating high-risk NMIBC. Recent trials of DV have predominantly focused on immunotherapy-related approaches. A phase Ib/II study (NCT04264936) investigating the effects of treatment with DV in combination with toripalimab (an ICI) in a subset of 10 mUC patients reported an overall response rate of 80% (via an interim analysis). Additionally, combining DV with tislelizumab (an ICI), has demonstrated satisfactory efficacy in patients with MIBC and NMIBC (39). The association of HER2+ with non-responsiveness to BCG therapy and with an immune-compromised state suggests that patients might benefit from initial treatment combining BCG therapy with HER2-targeted therapeutics, such as DV and ICIs, instead of BCG therapy alone. These findings provide a basis for combination therapy and support the theoretical rationale for using DV and ICIs in high-risk bladder cancer.

However, it is important to acknowledge some limitations of this study. First, since it is a retrospective study, there is an inherent risk of selection bias. Second, the sample size was relatively small, potentially limiting the generalizability of our findings. Furthermore, we did not directly investigate the mechanistic pathways through which HER2+ influences prognosis and treatment response. In bladder cancer, the frequent occurrence of HER2 protein overexpression, compared to HER2 gene amplification, is well-documented (40). This trend is similar to that observed for breast cancer, for which HER2-targeted therapeutics have been shown to be effective against tumors exhibiting gene amplification. Therefore, incorporating clinical inclusion criteria such as IHC-determined protein expression levels, along with investigating the gene mutations of *ERBB2*, may enhance our ability to effectively predict the response to BCG therapy, classify patients with a higher tendency for recurrence, particularly those in the class 2a category, and potentially identify candidates suitable for HER2-related therapeutics. However, the reliance solely on IHC methodology for this research represents another limitation of our study. Despite these limitations, our study demonstrated the potential of HER2+ as a molecular subtype, BCG response, and resistance mechanism within the TME of this patient group.

## 5 Conclusion

The HER2+ status of tumor samples predicted the response to BCG treatment, potentially providing alternative therapeutic strategies for the BCG non-responders. Additionally, TME trends of aggressive adaptive immune response stratified to HER2 provide insight into the mechanism of recurrence and BCG response. The HER2+ status observed more frequently in older age could be related to genetic subtype 2a, which is potentially associated with an aggressive adaptive immune system.

## Data availability statement

The original contributions presented in the study are included in the article/**Supplementary Material**. Further inquiries can be directed to the corresponding authors.

## Author contributions

WN: Conceptualization, Data curation, Formal analysis, Funding acquisition, Methodology, Project administration, Resources, Validation, Writing – original draft, Writing – review & editing, Investigation. HC: Conceptualization, Data curation, Formal analysis, Investigation, Methodology, Project administration, Validation, Writing – original draft, Writing – review & editing. YJ: Data curation, Formal analysis, Investigation, Writing – original draft, Writing – review & editing. HK: Data curation, Formal analysis, Writing – original draft, Writing – review & editing, Investigation. MP: Data curation, Formal Analysis, Writing – review & editing, Investigation, Software, Visualization. AC: Data curation, Formal analysis, Software, Visualization, Writing – review & editing, Funding acquisition, Methodology, Project administration, Supervision, Validation. JP: Data curation, Supervision, Writing – review & editing, Investigation, Resources. DE: Resources, Supervision, Writing – review & editing, Conceptualization, Investigation, Project administration. SK: Investigation, Methodology, Software, Validation, Visualization, Writing – original draft, Conceptualization, Project administration, Resources, Supervision, Writing – review & editing, Data curation, Formal analysis, Funding acquisition.

## Glossary

**Table:**

NMIBC	non-muscle invasive bladder cancer
BCG	Bacillus Calmette–Guérin
TURBT	transurethral resection of bladder tumor
MIBC	muscle invasive bladder cancer
EORTC	European Organization for Research and Treatment of Cancer
HER2	human epidermal growth factor receptor 2
CUETO	Spanish Urological Club for Oncological Treatment
HER2+	HER2 positive
EORTC-P	European Organization for Research and Treatment of Cancer Progression
EAU risk	European Association Urology risk group 2023
TME	tumor microenvironment
PFS	progression-free survival
OS	overall survival
EORTC-P	EORTC progression risk group
CIS	carcinoma in situ
EORTC-R	EORTC recurrence risk group
TMA	Tissue Microarray
IHC	immunohistochemical
PD-L1	programmed death-ligand 1
TILs	tumor-infiltrating lymphocytes
ICIs	immune checkpoint inhibitors
DV	Disitamab Vedotin
