# Supplementary material for: HER2 positivity predicts BCG unresponsiveness and adaptive immune cell exhaustion in EORTC risk-stratified cohort of bladder cancer
Source: Front Immunol. 2023 Dec 8;14:1301510. doi: 10.3389/fimmu.2023.1301510 (PMC10748406; doi:10.3389/fimmu.2023.1301510)
Supplement: Supplementary file 1 [file DataSheet_1.docx]

Supplementary Material

HER2 Positivity Predicts BCG Unresponsiveness and Adaptive Immune Cell Exhaustion in an EORTC Risk-Stratified Cohort of Bladder Cancer Patients

First Wook Nam1, Han Kyu Chae1, Yeonuk Jung2, Homin Kang2, Myungchan Park3, Ahnryul Choi4, Jong Yeon Park1, Dae-Woon Eom5†, and Sung Jin Kim1†

*** Correspondences:** Sung Jin Kim, MD, PhD, [bop1004@hanmail.net](mailto:bop1004@hanmail.net) and Dae-Woon Eom, MD, PhD, edwjyh@gnah.co.kr

# Supplementary Figures and Tables

## Supplementary Tables

Table S1. EORTC scoring system of weighing used to calculate disease recurrence and progression

| Factors | Recurrence | Progression |
| --- | --- | --- |
| No. of tumors |  |  |
| Single | 0 | 0 |
| 2–7 | 3 | 3 |
| ≥8 | 6 | 3 |
| Tumor size |  |  |
| <3 cm | 0 | 0 |
| ≥3 cm | 3 | 3 |
| Previous recurrence rate |  |  |
| Primary | 0 | 0 |
| ≤1 recurrence/year | 2 | 2 |
| >1 recurrence/year | 4 | 2 |
| T category |  |  |
| Ta | 0 | 0 |
| T1 | 1 | 4 |
| Carcinoma *in situ* |  |  |
| No | 0 | 0 |
| Yes | 1 | 6 |
| Grade |  |  |
| 1 | 0 | 0 |
| 2 | 1 | 0 |
| 3 | 2 | 5 |
| Total score | 0–17 | 0–23 |

Table S2. Notation of EORTC disease recurrence and progression according to total scores in this study

| Recurrence score(s) | EORTC recurrence risk group | Notation of recurrence risk group in this study |
| --- | --- | --- |
| 0 | Low risk | Low risk |
| 1–4 | Intermediate risk | Intermediate risk |
| 5–9 | Intermediate risk | High risk |
| 10–17 | High risk | Very high risk |
| Progression score(s) | EORTC Progression risk group | Notation of progression risk group in this study |
| 0 | Low risk | Low risk |
| 2–6 | Intermediate risk | Intermediate risk |
| 7–13 | High risk | High risk |
| 14–23 | High risk | Very high risk |

Table S3. Baseline clinicopathological results according to HER2 positive in BCG-treated patients

| Number of patients, n (%) | Total  67 (100) | HER2+  9 (13.4) | HER2−  58 (86.6) | *p*-value |
| --- | --- | --- | --- | --- |
| Age, year, median [IQR] | 71.0 [62.0–76.0] | 71.0 [65.0–76.0] | 70.0 [62.0–76.0] | 0.78 |
| Sex, n (%) |  |  |  | 1.00 |
| Male | 54 (80.6) | 7 (77.8) | 47 (81.0) |  |
| Female | 13 (19.4 ) | 2 (22.2) | 11 (19.0) |  |
| Tumor size (cm) |  |  |  | 1.00 |
| <3 | 38 (56.7) | 5 (55.6) | 33 (56.9) |  |
| ≥3 | 29 (43.3) | 4 (44.4) | 25 (43.1) |  |
| Number of tumors, n (%) |  |  |  | <0.05 |
| Single | 36 (53.7) | 1 (11.1) | 35 (60.3) |  |
| 2–7 | 29 (43.3) | 8 (88.9) | 21 (36.2) |  |
| ≥7 | 2 (3.0) | 0 (0.0) | 2 (3.4) |  |
| Tumor stage, n (%) |  |  |  | 1.00 |
| Ta | 26 (38.8) | 3 (33.3) | 23 (39.7) |  |
| T1 | 41 (61.2) | 6 (66.7) | 35 (60.3) |  |
| Tumor grade, n (%) |  |  |  | 0.34 |
| Low grade | 10 (14.9) | 0 (0.0) | 10 (17.2) |  |
| High grade | 54 (80.6) | 8 (88.9) | 46 (79.3) |  |
| Loss | 3 (4.5) | 1 (11.1) | 2 (3.4) |  |
| Concurrent CIS, n (%) | 8 (11.9) | 2 (22.2) | 6 (10.3) | 0.64 |
| EORTC-R risk score, n (%) |  |  |  | 0.08 |
| Intermediate (1–4) | 22 (32.8) | 0 (0.0) | 22 (37.9) |  |
| High risk (5–9) | 40 (59.7) | 8 (88.9) | 32 (55.2) |  |
| Very high (10–17) | 5 (7.5) | 1 (11.1) | 4 (6.9) |  |
| EORTC-P risk, n (%) | |  |  | 0.13 |
| Low (0) | 4 (6.0) | 0 (0.0) | 4 (6.9) |  |
| Intermediate (2–6) | 7 (10.4) | 0 ( 0.0) | 7 (12.1) |  |
| High risk (7–13) | 34 (50.7) | 4 (44.4) | 30 (51.7) |  |
| Very high (14–23) | 12 (17.9) | 4 (44.4) | 8 (13.8) |  |
| Recurrence, n (%) | 38 (56.7) | 8 (88.9) | 30 (51.7) | 0.08 |
| Progression, n (%) | 7 (10.4) | 1 (11.1) | 6 (10.3) | 1.00 |
| Expire event, n (%) | 22 (32.8) | 3 (33.3) | 19 (32.8) | 1.00 |
| Survival, months, median [IQR] | 89.0 [70.5–132.0] | 73.0 [50.0–137.0] | 89.5 [71.0–129.0] | 0.72 |

Table S3 presents the baseline clinicopathological characteristics of 67 BCG-treated patients according to HER2+. The results showed that 13.4% (9/67) of patients had HER2+, whereas the remaining 86.6% (58/67) did not. No significant differences were observed between the HER2+ and HER2- groups regarding age, sex, tumor size, tumor stage, tumor grade, and concurrent CIS. However, a significant difference was found in the number of tumors between the groups (*p* < 0.05), with a higher prevalence of multiple tumors in patients with HER2+. Furthermore, there was a trend towards higher EORTC-R risk scores in patients with HER2+, although it did not reach statistical significance (*p* = 0.079).

Table S4. Tumor microenvironment markers according to BCG treatment response

| Tumor microenvironment | Total  n = 67 | BCG non-responder | | | BCG responder  n = 29 | Total  Non-refractory  n = 54 | *p*-value* | *p*-value** |
| --- | --- | --- | --- | --- | --- | --- | --- | --- |
|  |  | Total  Non-responder  n = 38 | Refractory  n = 13 | Relapsed  n = 25 |  |  |  |  |
| HER2+, n (%) | 9 (13.4) | 8 (88.89) | 4 (44.44) | 4 (44.44) | 1 (11.11) | 5 (55.56) | 0.08 | 0.20 |
| PD-L1^+^, n (%) | 34 (51.5) | 19 (55.88) | 6 (17.65) | 13 (38.24) | 15 (44.12) | 28 (82.35) | 1.00 | 0.90 |
| PD1 [IQR] | 2.0 [ 0.0–12.5] | 2.5 [ 0.0–11.0] | 1.0 [ 0.0–18.0] | 3.0 [0.0–11.0] | 2.0 [0.0–13.0] | 2.5 [0.0–12.0] | 0.92 | 0.90 |
| CD8 [IQR] | 20.0 [ 5.5–46.0] | 19.0 [ 5.0–49.0] | 32.0 [ 4.0–49.0] | 18.0 [5.0–46.0] | 20.0 [8.0–36.0] | 19.0 [8.0–43.0] | 0.92 | 0.99 |
| KI67 [IQR] | 18.0 [ 6.0–30.0] | 15.5 [ 2.0–35.0] | 7.0 [ 0.0–35.0] | 18.0 [6.0–35.0] | 19.0 [11.0–25.0] | 18.5 [8.0–28.0] | 0.73 | 0.29 |

* Pearson’s χ2 test or Fisher’s exact test, BCG response vs. BCG non-response (two-tailed)

** Pearson’s χ2 test or Fisher’s exact test, BCG non-refractory vs. BCG refractory (two-tailed)

One patient value of PD-L1 included was missed. This patient was classified as a non-responder, relapsed.

Table S4 presents the tumor microenvironment in relation to BCG treatment response. Overall, 67 patients were analyzed, including BCG non-responders (n = 29) and BCG responders (n = 38), with further categorization into refractory (n = 13) and relapsed (n = 25) subgroups. The tumor microenvironment of HER2, PD-L1, PD1, CD8, and Ki67 was evaluated in these patients. Although no significant differences were observed in PD-L1, PD1, CD8, and Ki67 expression levels between BCG non-responders and responders, there was a trend towards increased HER2 expression in non-responders (*p* = 0.079).

Table S5. Association of EORTC recurrence risk groups and the tumor microenvironment in all patients

| Tumor microenvironment | Total (n = 160) | Low to intermediate risk (n = 79) | | High to very high risk (n = 81) | | *p*-value |
| --- | --- | --- | --- | --- | --- | --- |
|  |  | Low (n = 12) | Intermediate (n = 67) | High (n = 74) | Very high (n = 7) |  |
| HER2+, n (%) | 25 (15.6) | 11 (13.9) | | 14 (17.3) | | 0.71 |
|  |  | 0 (0.0) | 11 (16.4) | 13 (17.6) | 1 (14.3) | 0.48 |
| PD-L1+, n (%) | 80 (50.6) | 37 (47.4) | | 43 (53.8) | | 0.53 |
|  |  | 1 (8.3) | 36 (54.5) | 40 (54.8) | 3 (42.9) | <0.05 |
| PD1 median [IQR] | 1.0 [ 0.0–9.0] | 0.0 [ 0.0– 6.0] | | 3.0 [ 0.0–12.0] | | <0.01 |
|  |  | 0.0 [0.0–0.0] | 0.0 [0.0–9.0] | 3.0 [0.0–12.0] | 2.0 [0.0–12.0] | <0.01 |
| CD8 median [IQR] | 16.0 [ 8.0–37.0] | 15.0 [5.0–33.5] | | 20.0 [13.0–46.0] | | <0.05 |
|  |  | 12.5 [10.0–19.3] | 18.0 [9.0–38.0] | 15.0 [4.0–37.0] | 17.0 [11.0–46.0] | 0.63 |
| Ki67 median [IQR] | 13.0 [4.0–26.0] | 12.0 [3.0–22.0] | | 26.0 [18.0–39.0] | | <0.001 |
|  |  | 4.0 [1.0–9.8] | 12.0 [5.0–22.0] | 18.0 [4.0–35.0] | 17.0 [3.0–28.0] | <0.001 |

presents the distribution of TME biomarkers (PD1, CD8, and Ki67) among 160 patients, stratified based on EORTC recurrence risk as follows: low (n = 12), intermediate risk (n = 67), high risk (n = 74) and very high risk (n = 7).

The results reveal no significant difference in the frequency of HER2+ expression between the low-to-intermediate risk and high-to-very high-risk groups (*p* = 0.708). However, significant differences were observed in the levels of PD1 (*p* = 0.006), CD8 (*p* = 0.013), and Ki67 (*p* < 0.001) between the two risk groups. Patients in the high-to-very high-risk group exhibited higher levels of these biomarkers than those in the low-to-intermediate risk group. The expression of PD-L1 showed no significant difference between the overall risk groups (*p* = 0.5271); however, a remarkable difference was observed when comparing subcategories (*p* = 0.0216).

Table S6. Association of EORTC progression risk groups and the tumor microenvironment in all patients

| Tumor microenvironment | Total (n = 149) | Low to intermediate risk (n = 71) | | High to very high risk (n = 78) | | *p*-value |
| --- | --- | --- | --- | --- | --- | --- |
|  |  | Low (n = 28) | Intermediate (n = 43) | High (n = 60) | Very high (n = 18) |  |
| HER2+, n (%) | 24 (16.1) | 7 (9.9) | | 17 (21.8) | | 0.08 |
|  |  | 0 (0.0) | 7 (16.3) | 11 (18.3) | 6 (33.3) | <0.05 |
| PD-L1^+^, n (%) | 72 (49.0) | 26 (37.1) | | 46 (59.7) | | <0.05 |
|  |  | 7 (25.9) | 19 (44.2) | 37 (61.7) | 9 (52.9) | <0.05 |
| PD1 median [IQR] | 1.0 [0.0– 8.5] | 0.0 [0.0–1.0] | | 4.5 [0.0–13.0] | | <0.001 |
|  |  | 0.0 [0.0–1.0] | 0.0 [0.0–2.0] | 4.0 [0.0–12.5] | 7.0 [1.0–13.0] | <0.001 |
| CD8 median [IQR] | 16.0 [8.0–35.0] | 13.0 [5.0–24.5] | | 17.5 [8.0–41.0] | | <0.05 |
|  |  | 14.5 [9.0–28.5) | 12.0 [5.0–23.0) | 17.5 [8.25–37] | 18.0 [4.8–61.5] | 0.16 |
| Ki67 median [IQR] | 13.0 [4.0–26.0] | 8.0 [2.5–13.0] | | 22.5 [12.0–38.0] | | <0.001 |
|  |  | 7.5 [3.0–12.0] | 8.0 [2.0–15.0] | 18.0 [7.8–32.0] | 34.5 [25.8–54.0] | <0.001 |

Table S6 presents the distribution of tumor microenvironment biomarkers (PD1, CD8, and Ki67) among 149 patients, according to EORTC-P risk as follows: low (n = 28), intermediate risk (n = 43), high risk (n = 60) and very high risk (n = 18).

The tumor microenvironment of HER2+, PD-L1+, PD1, CD8, and Ki67 were analyzed according to EORTC-P and their potential role in the tumor microenvironment. The results demonstrated a marginally significant difference in HER2+ between the low-to-intermediate risk and high-to-very high-risk groups (*p* = 0.079). When examining the subcategories of EORTC-P, a significant difference was observed in HER2+ (*p* < 0.05). Furthermore, a significant difference was found in the proportion of patients with PD-L1+ between the two risk groups (*p* < 0.05), as well as within subcategories (*p* < 0.05). PD1 (*p* < 0.001), CD8 (*p* < 0.05), and Ki67 (*p* < 0.001) showed significant differences between the low-to-intermediate risk and high-to-very high-risk groups. Patients in the high-to-very high-risk group exhibited higher levels of these tumor microenvironment biomarkers than those in the low-to-intermediate risk group. These findings suggest that the tumor microenvironment varies among patients with different EORTC-P risks.

Table S7. Association of HER2 positive expression and tumor microenvironment in all patients

| Tumor microenvironment | Total (n = 160) | HER2− (n = 135) | | HER2+ (n = 25) | | *p*-value |
| --- | --- | --- | --- | --- | --- | --- |
|  |  | HER2 0 (n = 99) | HER2 1+ (n = 36) | HER2 2+ (n = 16) | HER2 3+ (n = 9) |  |
| PD-L1^+^, n (%) | 80 (50.6) | 61 (45.9) | | 19 (76.0) | | <0.05 |
|  |  | 44 (44.9) | 17 (48.6) | 10 (62.5) | 9 (100.0) | <0.05 |
| PD1 median [IQR] | 1.0 [ 0.0– 9.0] | 0.0 [ 0.0– 9.0] | | 6.0 [1.0–10.0] | | <0.05 |
|  |  | 0.0 [0.0–8.0] | 3.0 [0.0–11.0] | 3.0 [0.0–7.0] | 10.0 [4.0–21.5] | <0.05 |
| CD8 median [IQR] | 16.0 [ 8.0–37.0] | 15.0 [5.0–33.5] | | 20.0 [13.0–46.0] | | <0.05 |
|  |  | 15.0 [6–33] | 13.5 [2.5–36.3] | 16.5 [12.0–42.8] | 41.0 [19.5–69.0] | <0.05 |
| Ki67 median [IQR] | 13.0 [4.0–26.0] | 12.0 [ 3.0–22.0] | | 26.0 [18.0–39.0] | | <0.001 |
|  |  | 9 [2–17] | 22.5[11.3–36.5] | 23.5[16.5–37.8] | 30.0 [17.0–51.0] | <0.001 |

Table S7 presents the distribution of TME biomarkers (PD1, CD8, and Ki67) among 160 patients stratified according to HER2 expression. The participants were categorized into HER2^-^ (n = 135) and HER2+ (n = 25) groups and further stratified based on their HER2 expression levels (HER2 0 (n = 99), HER2 1+ (n = 36), HER2 2+ (n = 16), and HER2 3+ (n = 9)).

A significant difference was found in PD-L1 expression between HER2^-^ and HER2+ groups (p = 0.011), as well as among the various HER2 expression levels (p = 0.0116). Patients with HER2+ tumors had a higher proportion of PD-L1. Furthermore, the HER2+ group exhibited significantly higher levels of PD1 (*p* = 0.027), CD8 (*p* = 0.013), and Ki67 (*p* < 0.001) than the HER2^-^ group. The same trend was observed across the different HER2 expression levels.

Table S8. Association of EORTC recurrence risk groups and tumor microenvironment in BCG-treated patients

| Tumor microenvironment | Total (n = 67) | EORTC recurrence risk group | | | *p*-value |
| --- | --- | --- | --- | --- | --- |
|  |  | Intermediate (n = 22) | High (n = 40) | Very high (n = 5) |  |
| HER2+, n (%) | 9 (13.2) | 0 (0.0) | 8 (20.0) | 1 (20.0) | 0.08 |
| PD-L1^+^, n (%) 66 | 34 (52.2) | 10 (45.5) | 21 (53.8) | 3 (60.0) | 0.76 |
| PD1, median [IQR] | 2.0 [0.0–12.5] | 0.0 [0–12.5] | 4.5 [0.0–13.0] | 7.0 [0.0–13.0] | 0.19 |
| CD8, median [IQR] | 20.0 [5.5–46.0] | 14.5 [4.8–60.5] | 20.5 [5.3–44.0] | 20 [11.5–62.0] | 0.85 |
| Ki67, median [IQR] | 18.0 [6.0–30.0] | 12.0 [7.5–27.5] | 18.5 [4.5–34.8] | 17.0 [2.5–26.5] | 0.83 |

Table S8 presents the distribution of tumor microenvironment biomarkers (PD1, CD8, and Ki67) in BCG-treated patients, stratified according to their EORTC-R risk groups as follows: intermediate (n = 22), high (n = 40), and very high (n = 5). HER2+, although not statistically significant (*p* = 0.079), appeared to be more prevalent in the high (20.0%) and very high (20.0%) recurrence risk groups than in the intermediate group (0.0%). PD-L1+ showed no significant differences among the groups (p = 0.76). No significant differences were observed in PD1 (*p* = 0.19), CD8 (*p* = 0.85), and Ki67 (*p* = 0.83) levels according to EORTC-R risk groups.

Table S9. Association of EORTC progression risk groups and tumor microenvironment in BCG-treated patients

| Tumor microenvironment | Total (n = 57) | Low to intermediate (n = 11) | | High to very high (n = 46) | | *p*-value |
| --- | --- | --- | --- | --- | --- | --- |
|  |  | Low (n = 4) | Intermediate (n = 7) | High (n = 34) | Very high (n = 12) |  |
| HER2+, n (%) | 8 (14.0) | 0 (0.0) | | 8 (17.4) | | 0.20 |
|  |  | 0 (0.0) | 0 (0.0) | 4 (11.8) | 4 (33.3) | 0.13 |
| PD-L1^+^, n (%) | 27 (48.2) | 3 (27.3) | | 24 (53.3) | | 0.23 |
|  |  | 1 (25.0) | 2 (28.6) | 18 (52.9) | 6 (54.5) | 0.49 |
| PD1 median [IQR] | 2.0 [0.0–12.0] | 0.0 [0.0– 0.0] | | 4.5 [0.0–13.0] | | <0.01 |
|  |  | 0.0 [0.0–0.8] | 0.0 [0.0–0.0] | 3.0 [0.0–13.5] | 10.0 [1.5–13.75] | <0.01 |
| CD8 median [IQR] | 18.0 [5.0–38.0] | 13.0 [ 3.5–21.0] | | 21.0 [ 6.0–49.0] | | 0.17 |
|  |  | 20.5 [3.5–74.3] | 13.0 [2.0–16.0] | 21.0 [5.8–39.3] | 29.0 [6.5–67.5] | 0.30 |
| Ki67 median [IQR] | 18.0 [6.0–32.0] | 9.0 [2.5–12.0] | | 21.0 [7.0–35.0] | | <0.05 |
|  |  | 8.5 [4.3–11.3] | 9.0 [1.0–12.0] | 17.0 [4.0–26.75] | 33.5 [25.3–42.0] | <0.001 |

Table S9 presents the distribution of tumor microenvironment biomarkers in 57 BCG-treated patients, stratified based on their EORTC-P risk groups as follows: low (n = 4), intermediate risk (n = 7), high risk (n = 34), and very high risk (n = 12). Although not statistically significant, HER2+ was more frequent in the high-to-very high-risk group (17.4%) than in the low-to-intermediate group (0.0%, *p* = 0.195). PD-L1+ also showed no significant difference between the two groups (*p* = 0.225). However, the PD1 level was significantly different (*p* < 0.01), with higher levels in the high-to-very high-risk group. CD8 level showed no significant differences between the groups (*p* = 0.169). Ki67 levels were significantly higher in the high-to-very high-risk group (*p* < 0.05).

Table S10. Association of HER2 expression and tumor microenvironment in BCG-treated patients

| Tumor microenvironment | Total (n = 57) | HER2− (n = 58) | | HER2+ (n = 9) | | *p*-value |
| --- | --- | --- | --- | --- | --- | --- |
|  |  | HER2 0 (n = 37) | HER2 1+ (n = 21) | HER2 2+ (n = 5) | HER2 3+ (n = 4) |  |
| PD-L1^+^, n (%) | 8.0 (14.0) | 27 (56.6) | | 7 (77.8) | | 0.17 |
|  |  | 17 (46.0) | 10 (50.0) | 3 (60.0) | 4 (100.0) | 0.22 |
| PD1 median [IQR] | 2.0 [ 0.0–12.0] | 0.0 [ 0.0–0.0] | | 4.5 [ 0.0–13.0] | | <0.01 |
|  |  | 0.0 [0.0–0.8] | 0.0 [0.0–0.0] | 3.0 [0.0–13.5] | 10.0 [1.5–13.8] | <0.01 |
| CD8 median [IQR] | 20.0 [ 5.5–46.0] | 16.0 [ 5.0–43.0] | | 36.0 [18.0–66.0] | | 0.09 |
|  |  | 15.0 [5.5–42.0] | 21.0 [2.0–46] | 18.0 [14.5–56.0] | 54.5 [24.0–82.0] | 0.31 |
| Ki67 median [IQR] | 18.0 [6.0–30.0] | 16.5 [6.0–25.0] | | 34.0 [21.0–39.0] | | 0.07 |
|  |  | 11 [2.5–21.5] | 23 [14.5–36] | 34 [12–42] | 32 [6.5–63.5] | <0.05 |

Table S10 presents the distribution of tumor microenvironment biomarkers in 57 BCG-treated patients stratified according to HER2 expression. The tumor microenvironment of PD-L1+, PD1, CD8, and Ki67 was evaluated for their association with HER2 expression, which was categorized as HER2 0 (n = 37), HER2 1+ (n = 21), HER2 2+ (n = 5), and HER2 3+ (n = 4). The results demonstrated that PD1 expression was significantly higher in patients with HER2+ (*p* < 0.01), with those with HER2 3+ showing the highest PD1 expression. Higher Ki67 expression was observed in patients with increased HER2 expression (*p* < 0.05).

## Supplementary Figures


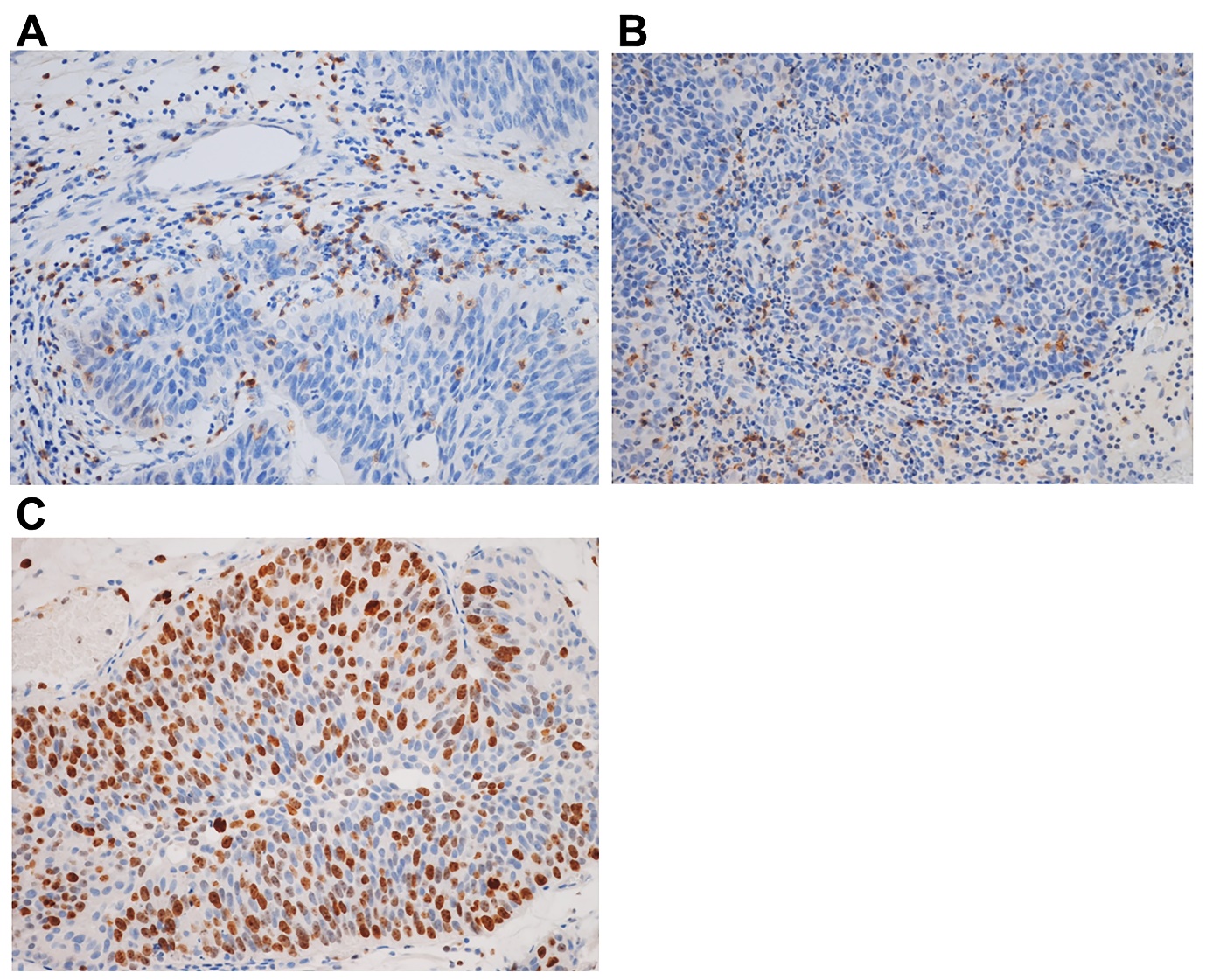


Figure S1. Immunohistochemical staining of PD-1, CD8, and Ki-67 at 200× magnification.

(A) Tumor cells showing PD-1+ T-cell infiltration in the tumor epithelium and stroma. (B) Tumor cells showing CD8+ T-cell infiltration in the tumor epithelium and stroma. (C) Tumor cells showing high Ki-67 labeling index (>50%).

**
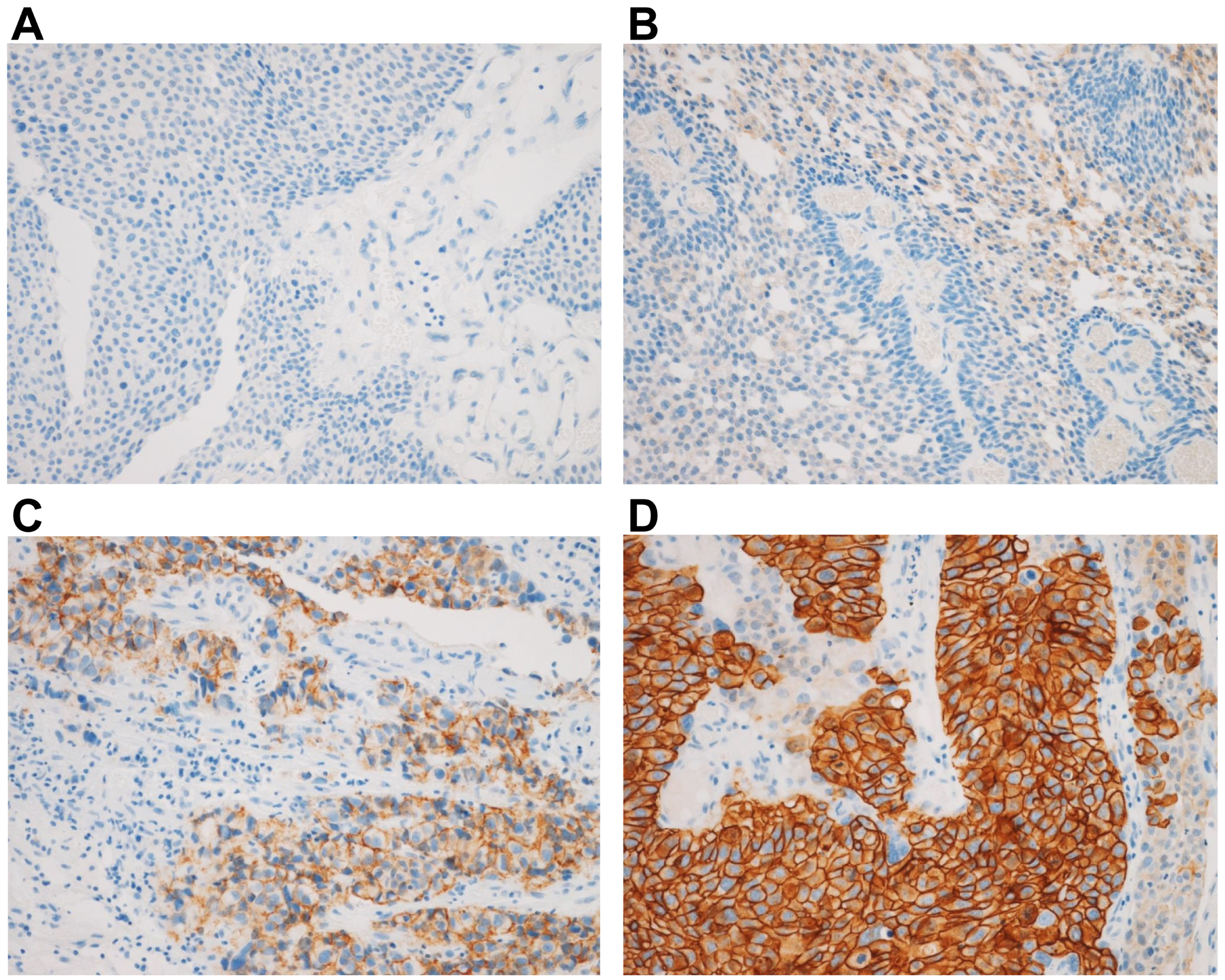
**

Figure S2. Immunohistochemical staining showing HER2 expression at 200× magnification.

(A) HER2− included ‘‘HER2 0’’ and ‘‘HER2 1+.’’ HER2 0: no staining (IHC score 0); (B) HER2 1+: weak or partial membrane staining in ≤10% tumor cells (immunohistochemical [IHC] score, 1). (C) HER2+ (HER2 positive) included ‘‘HER2 2+’’ and ‘‘HER2 3+.’’ HER2 2+ indicates weak or moderate complete membrane staining in >10% tumor cells (IHC score, 2); (D) HER2 3+ indicates strong complete membrane staining in >10% tumor cells (IHC, score 3).
